# Additive effects of narrowband light and optical defocus on chick eye growth and refraction

**DOI:** 10.1186/s40662-023-00332-7

**Published:** 2023-04-01

**Authors:** Rachel Ka-man Chun, Kit-ying Choy, King-kit Li, Thomas Chuen Lam, Dennis Yan-yin Tse, Chi-ho To

**Affiliations:** 1 Laboratory of Experimental Optometry, Centre for Myopia Research, School of Optometry, The Hong Kong Polytechnic University, Kowloon, Hong Kong; 2 Research Centre for SHARP Vision (RCSV), The Hong Kong Polytechnic University, Kowloon, Hong Kong; 3 Centre for Eye and Vision Research (CEVR), 17W Hong Kong Science Park, New Territories, Hong Kong

**Keywords:** Light, Optical defocus, Myopia, Wavelength, Chicks

## Abstract

**Background:**

In the past decade and during the COVID pandemic, the prevalence of myopia has reached epidemic proportions. To address this issue and reduce the prevalence of myopia and its complications, it is necessary to develop more effective interventions for controlling myopia. In this study, we investigated the combined effects of narrowband lights and competing defocus on eye growth and refraction in chicks, an important step in understanding the potential for these interventions to control myopia. This is the first time these effects have been characterized.

**Methods:**

Three groups of five-day-old chicks (n = 8 per group) were raised in three different lighting conditions: white, red, and blue for 13 days in a 12/12-h light/dark diurnal cycle. One eye was randomly selected for applications of a dual-power optical lens (− 10 D/ + 10 D, 50∶50), while another eye was left untreated as control. Vitreous chamber depth (VCD), axial length (AL), choroidal thickness (CT) and refractive errors were measured at pre-exposure (D0) and following 3 (D3), 7 (D7), 10 (D10), and 13 days (D13) of light exposure.

**Results:**

Under white light, the dual-power lens induced a hyperopic shift [at D13, mean spherical equivalent refraction (SER), treated *vs.* control: 4.81 ± 0.43 D *vs.* 1.77 ± 0.21 D, *P* < 0.001] and significantly reduced the progression of axial elongation (at D13, change in AL, treated *vs.* control: 1.25 ± 0.04 mm *vs.* 1.45 ± 0.05 mm, *P* < 0.01). Compared to white light alone, blue light alone induced a hyperopic shift (at D13, mean SER, blue *vs.* white: 2.75 ± 0.21 D *vs.* 1.77 ± 0.21 D, *P* < 0.01) and significantly reduced axial elongation (at D13, change in AL, blue *vs.* white: 1.17 ± 0.06 mm *vs.* 1.45 ± 0.05 mm, *P* < 0.01) in control eyes. When comparing all conditions, eyes exposed to blue light plus dual-power lens had the least axial elongation (at D13, change in AL, 0.99 ± 0.05 mm) and were the most hyperopic (at D13, mean SER, 6.36 ± 0.39 D).

**Conclusions:**

Both narrowband blue light and dual-power lens interventions were effective in inducing a hyperopic shift in chicks, and provided protection against myopia development. The combination of these interventions had additive effects, making them potentially even more effective. These findings support the use of optical defocus interventions in combination with wavelength filters in clinical studies testing their effectiveness in treating myopia in children.

**Supplementary Information:**

The online version contains supplementary material available at 10.1186/s40662-023-00332-7.

## Background

Myopia is a condition in which the eyeball becomes excessively elongated, leading to blurry distance vision because the projected image is focused in front of the retina. The prevalence of myopia has reached epidemic levels in the past decade and during the COVID pandemic [1–3], particularly in East Asian countries such as China and Singapore. Approximately 50% of the world population is predicted to become myopic by the year 2050, of which nearly 10% would comprise high myopia individuals [4]. Myopia, particularly high myopia, increases the risk of sight-threatening diseases such as glaucoma [5, 6] and retinal degeneration [7, 8]. While blurry distance vision can be corrected with optical or surgical methods, it is important to address myopia itself to reduce the risk of these complications. The financial costs to public health systems due to high myopia-related complications are significant. Implementing myopia control interventions in schoolchildren is the most practical method for reducing the prevalence of sight-threatening diseases in the coming years and decades.

Optical intervention is commonly used for myopia control in schoolchildren. For myopic defocus, a positive powered lens casts the optical image in front of the retina [9, 10]. Myopic defocus has been shown to inhibit the development of myopia in animal models such as chicks [11–13], guinea pigs [14–16], tree shrews [13, 17], and rhesus monkeys [13, 18–20]. Myopic defocus-induced reduction in myopia development was also associated with reduced vitreous chamber depth (VCD) and axial length (AL), and a significant choroidal thickening [14, 21]. In animal models wearing optical lenses with competing optical defocus (myopic and hyperopic), myopic defocus is the more potent and dominant stimulus for eye growth [22, 23].

In children, myopic defocus is typically incorporated in the periphery of otherwise negatively powered corrective lenses, such that the central vision is not affected by the myopic defocus [24–27]. Recent studies have showed that dual-focus lens can effectively slow myopia progression and axial elongation in children [25, 28–31]. Dual-focus lens has a central zone for distant refractive correction and concentric rings as peripheral zone, which provides additional positive power, alternating with the normal distance correction [25, 29]. Full-time wearing of dual-focus soft contact lens also slowed myopia progression and axial elongation [25, 28, 29, 31, 32]. The effect was sustainable and may further slow myopia progression in subsequent full-time lens wearing period [25, 28, 30]. The protective effect was more effective for lens with high add power (+ 2.0 D) when compared with medium add power (+ 1.5 D) [31]. Therefore, using opposing dual-power lenses (− 10 D/ + 10 D, 50:50) in animal models is a better approximation of the myopic defocus administered to schoolchildren when compared with lenses that are fully positively powered at + 10 D [25].

Prior to or in addition to optical interventions for myopia in schoolchildren, clinicians recommend increasing the time spent outdoors due to its protective effect against myopia onset and progression [33–36]. The protective effect of increased time outdoors is mainly reliant on the exposure to the high level of sunlight but independent of the physical activities performed [35, 37]. Children with longer durations of exposure to moderate light intensities (1000 lx or more) outdoors are protected against myopia [38]. Continuous near work without rest and reduced time of outdoor activities increase the risk of myopia prevalence in children [37, 39]. Children spending more time outdoors are less likely to become myopic [33, 35, 37, 38, 40, 41], regardless of how much time they spend doing near work [34]. The exact mechanism through which outdoor activities reduce myopia prevalence and progression remains ill-defined. However, light intensity and wavelength spectrum have been suggested to play a role. Outdoor light intensity is much stronger relative to indoor light, and bright light reduced myopia development in animal models, including chicks [42–44], guinea pigs [45], rhesus monkeys [46, 47], and mice [48]. In our previous study, we demonstrated an interaction between bright light and competing defocus in chicks [49]. We found that the inhibitory effects of bright light, and myopic defocus on eye growth in chicks were additive.

Recent studies have demonstrated that repeated low-level long wavelength red light therapy could effectively control myopia progression in children [50–52]. The effect of repeated short exposure of long wavelength (635 nm [52] or 650 nm [50, 51]) at low intensity (up to 2 mW) for three minutes, twice per day, was promising. Over the 12-month study period, it slowed 76.6% myopia progression and reduced 69.4% axial elongation [50]. On the other hand, daily wear of violet light transmitting spectacles lens showed significant reduction in axial elongation in children over a one-year period [53]. Moreover, the implantation of violet light transmitting phakic intraocular lens significantly slowed axial elongation in adults with high myopia (less than − 10 D) over a five-year period [54]. The effects of short wavelength light exposure on myopia control in animal models and clinical studies are further explored in discussion section. However, there is a lack of literature exploring the interaction between light wavelength spectrum and competing defocus on ocular development. This study aimed to quantitatively characterize the combined effect of specific spectrum of light and competing defocus on eye growth in chicks.

## Methods

### Animal and study design

The care and use of the chicks were performed according to the Association for Research in Vision and Ophthalmology Statement for the Use of Animals in Ophthalmic and Vision Research. The experimental protocols (No. 17-18/32-SO-R-OTHERS) complied with university guidelines and were approved by the Animal Subjects Ethics Sub-committee of The Hong Kong Polytechnic University. Animals had ad libitum access to food and water.

**Fig. 1:**
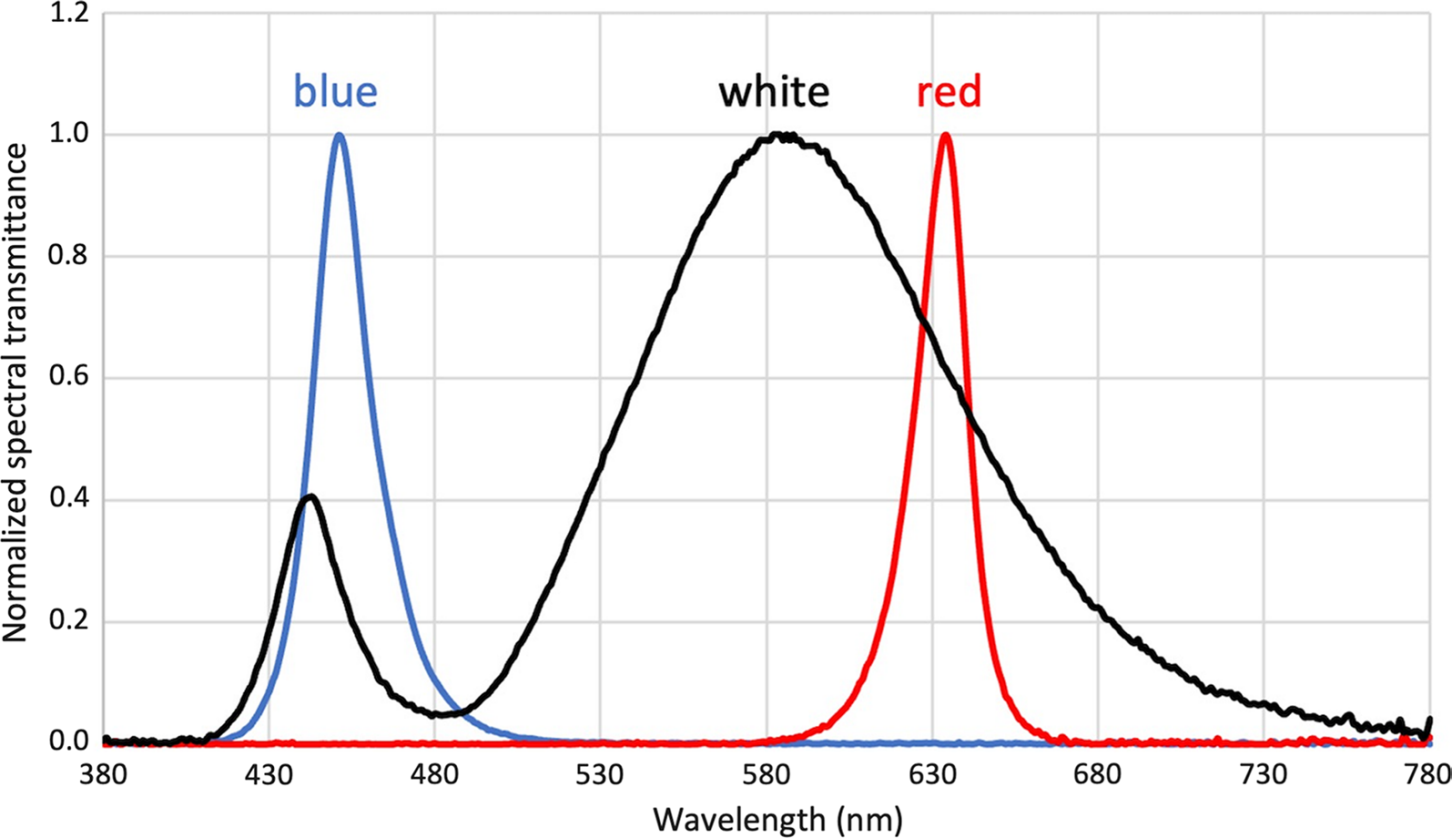
Normalized spectral transmittance of three types of lightings used for chick rearing. Red lighting had a sharp peak at 634 ± 15 nm and blue lighting at 451 ± 15 nm. White lighting had a broad spectrum with a high peak at 586 nm and a lower peak at 443 nm

**Fig. 2:**
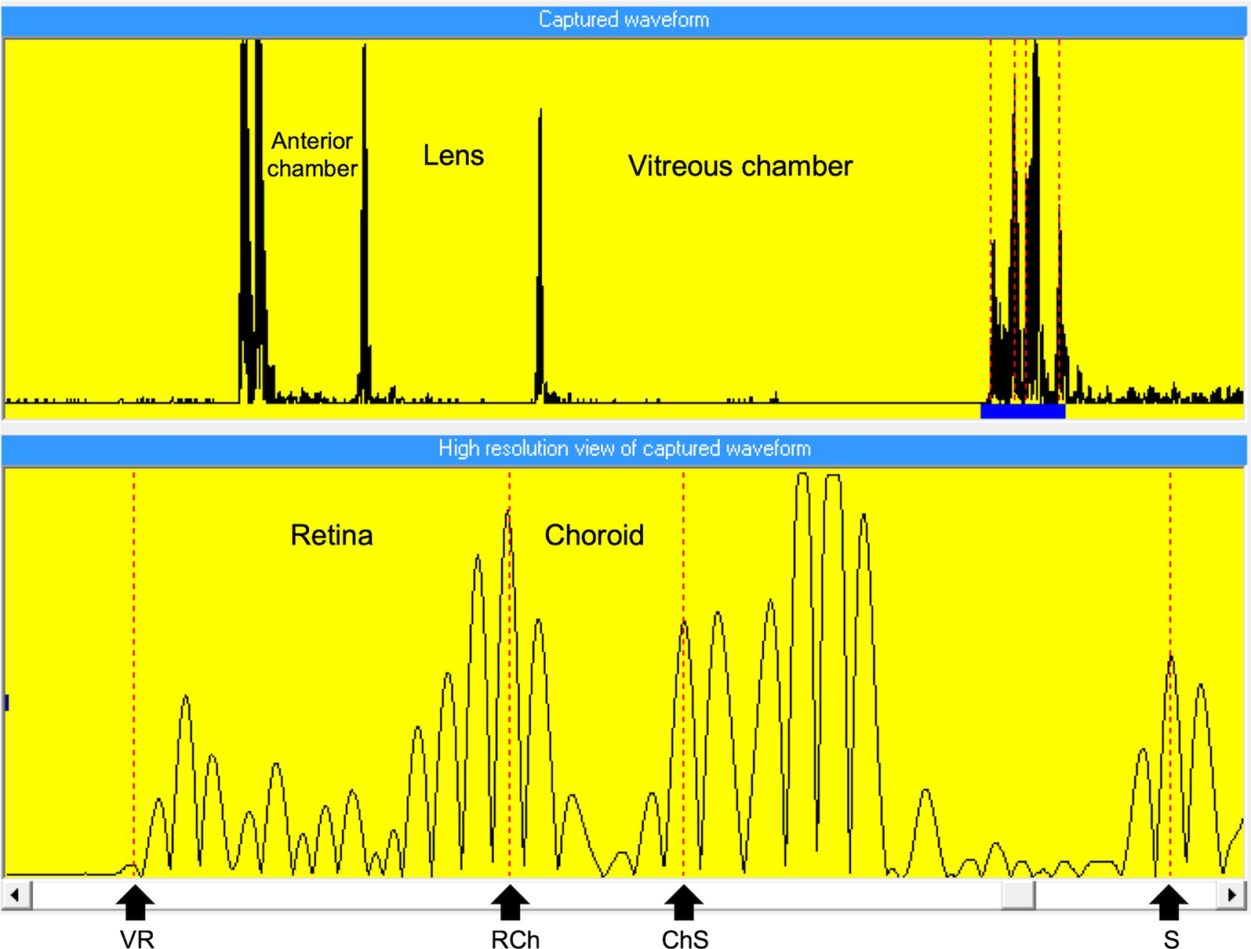
A representative ultrasound trace obtained by A-scan ultrasonography. The upper panel shows the captured ultrasound trace. The bottom panel shows the expanded trace of the back of eyes (highlighted by a horizontal blue bar in the upper panel) to illustrate the selected peaks corresponding to the interfaces: vitreal-retinal (VR) interface, peak in front of retina; retinal-choroidal (RCh) interface, peak in front of choroid; choroidal–scleral (ChS) interface, peak at inner sclera; and the back of sclera (S), peak at outer sclera

There were three groups of five-day-old White Leghorn chicks (*Gallus gallus domesticus*) (n = 24, 8 per group). They were bred from specific-pathogen-free eggs (Jinan SPAFAS Poultry Co., Jinan, China) in the Centralised Animal Facilities of The Hong Kong Polytechnic University. The chicks were raised in three different lighting conditions: white (W), red (R), and blue (B) for 13 days in a 12/12-h light/dark diurnal cycle. Six of 3-W light emitting diode (LED) light bulbs (UMAKED, Guangdong, China) were attached to the roof of the cage and produced narrowband red (634 ± 15 nm) or blue (451 ± 15 nm) light or a broadband white light depending on the conditions. The spectrum for each lighting condition is shown in Fig. 1. To control the light brightness, the luminance in each lighting condition was set to be approximately 250 lx. The luminance of light condition was measured by cal-Light 400 Digital Light Meter (Cooke Corporation, Romulus, MI, USA). Refractive errors and ocular parameters were measured at the following timepoints relative to the start of controlled light exposure: pre-exposure (D0) and following 3 (D3), 7 (D7), 10 (D10), and 13 days (D13) of exposure. All measurements were done at the same time of the day, between 9 a.m. to 11 a.m., to minimize the effects of diurnal variation on ocular parameters. The refractive errors were measured by BETA 200 Streak Retinoscope (HEINE, Herrsching, Germany), while the ocular parameters were measured by high frequency A-scan ultrasonography (Panametrics, Waltham, MA, USA) with a 30 MHz transducer at a rate of 100 MHz. Because choroidal thickness (CT) was negatively correlated with eye growth in chicks [10, 55], CT was also measured with ultrasound and a custom-written algorithm to identify peaks corresponding to vitreal-retinal (VR) interface, the retina-choroidal (RCh) interface and the choroidal-scleral (ChS) interface and the back of the sclera (S) (see Fig. 2) [56].

**Fig. 3:**
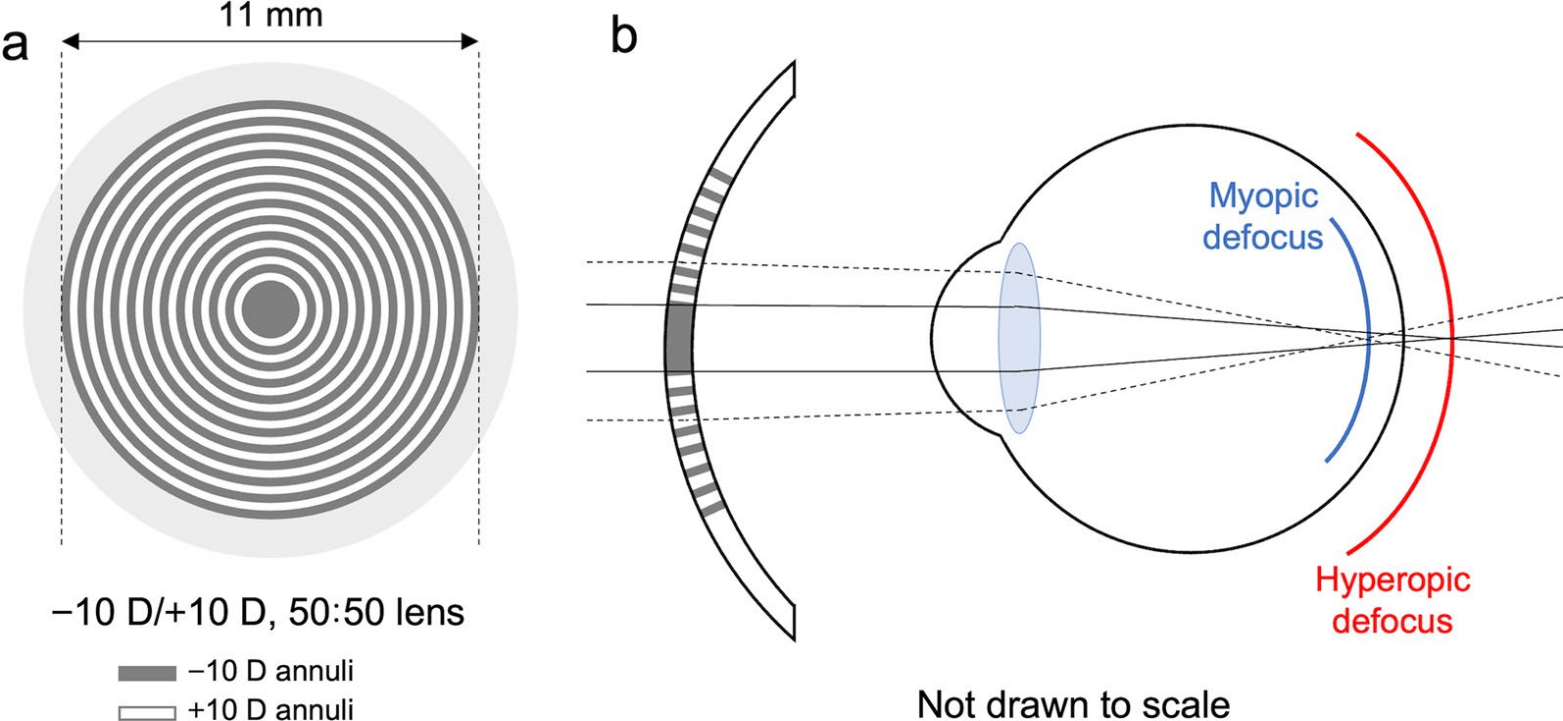
Dual-power optical lens. **a** A schematic diagram showing the dual-power lens (− 10 D/ + 10 D, 50:50). The lens is concentric with alternating rings with + 10 D and − 10 D power. **b** A schematic diagram showing the two distinct image planes induced by the − 10 D/ + 10 D, 50:50 dual-power lens

After the baseline measurement of ocular parameters and refraction, one eye was randomly selected for applications of a dual-power optical lens (− 10 D/ + 10 D, 50:50), while another eye was left untreated as control. The concept and configuration of the dual-power optical power have been described previously [22, 49]. The lens was designed using an optical design program (Zemax; Zemax Design Corp., Bellevue, WA) based on the Fresnel’s principle and optimized to minimize spherical aberrations [22]. Lenses were manufactured from polymethylmethacrylate (PMMA) cast molding by the State Key Laboratory of Ultra-precision Machining Technology in The Hong Kong Polytechnic University. The optical zone of the lenses had a diameter of 11 mm and the extend of the field of view through it was approximately 150° [22]. The lenses had an anterior radius of curvature of 6.68 mm and were concentric with alternating rings with + 10 D and − 10 D defocus (Fig. 3a). The central zone of the lenses had a − 10 D power, and the pitch width of each annulus was 0.1 mm [49]. The multizone dual-power lenses produce two distinct image planes, in which the − 10 D rings produce a hyperopic defocus while the + 10 D rings produce a myopic defocus on the retina (Fig. 3b). Body weight was also monitored before and after 13 days of lens wear.

### Data analysis

AL was defined as the sum of components from anterior cornea to anterior retina. Refractive errors were presented as spherical equivalent refraction (SER). SER is the sum of spherical power and half of the cylindrical power measured by retinoscopy. The interocular difference (IOD) in refractive errors and ocular parameters was assessed by measuring the parameters in chicks under each light condition and then subtracting the measurements in control eyes from that in lens-wearing eyes. Changes were then compared using one-way repeated-measures ANOVA with Tukey HSD post hoc test or two-way repeated-measures ANOVA with Bonferroni post hoc test. Data are presented as mean change ± standard error of mean (SEM). A *P* value of less than 0.05 was considered statistically significant. The above analyses were performed using GraphPad Prism (version 8.0, GraphPad Software, San Diego, California, USA, www.graphpad.com).

## Results

### Baseline measurement

At baseline, there was no significant difference between treatment groups in ocular parameters (Table 1).

**Table 1:** Baseline ocular parameters and refractive errors between different groups

Parameter	ACD (mm)	LT (mm)	VCD (mm)	AL (mm)	SER (D)	CT (µm)
Red with lens	1.35 ± 0.02	1.95 ± 0.02	5.23 ± 0.05	8.53 ± 0.04	3.84 ± 0.08	192.13 ± 11.78
Red without lens	1.36 ± 0.02	1.91 ± 0.02	5.25 ± 0.05	8.53 ± 0.05	3.81 ± 0.09	183.33 ± 9.62
IOD	− 0.01 ± 0.01	0.03 ± 0.02	− 0.02 ± 0.03	0.00 ± 0.03	0.03 ± 0.13	8.79 ± 10.29
Blue with lens	1.37 ± 0.01	1.95 ± 0.03	5.32 ± 0.02	8.65 ± 0.04	3.66 ± 0.31	183.06 ± 8.45
Blue without lens	1.38 ± 0.03	1.90 ± 0.01	5.35 ± 0.04	8.63 ± 0.06	3.56 ± 0.26	200.48 ± 11.97
IOD	− 0.01 ± 0.02	0.06 ± 0.04	− 0.03 ± 0.03	0.02 ± 0.03	0.09 ± 0.26	− 15.42 ± 10.14
White with lens	1.35 ± 0.01	1.94 ± 0.01	5.23 ± 0.05	8.51 ± 0.06	3.72 ± 0.17	171.06 ± 8.17
White without lens	1.35 ± 0.01	1.93 ± 0.01	5.23 ± 0.04	8.52 ± 0.05	3.56 ± 0.15	163.83 ± 9.80
IOD	− 0.01 ± 0.01	0.01 ± 0.01	− 0.01 ± 0.02	− 0.01 ± 0.02	0.16 ± 0.12	7.23 ± 8.77

Two-way repeated-measures ANOVA with Bonferroni post hoc test was performed to compare the results. Data represents mean ± SEM (n = 8 per condition). *ACD* = anterior chamber depth; *AL* = axial length; *CT* = choroidal thickness; *IOD* = interocular difference; *LT* = lens thickness; *SER* = spherical equivalent refraction; *VCD* = vitreous chamber depth

### Effect of opposing dual-power lenses on eye growth

**Fig. 4:**
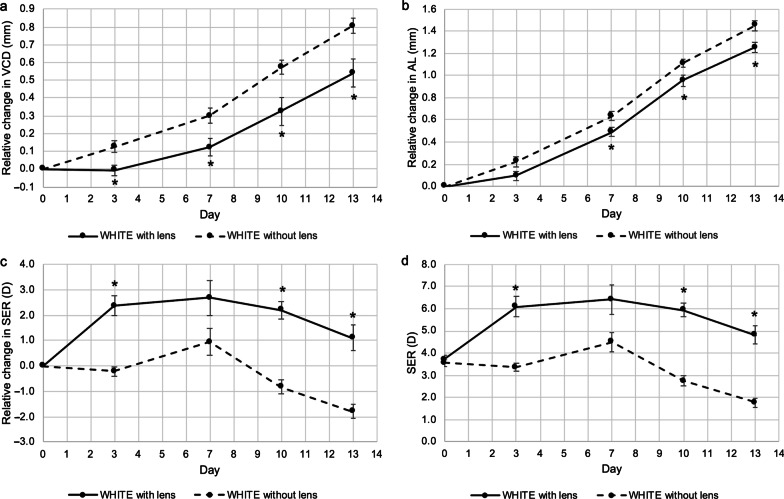
Effect of opposing dual-power lenses on eye growth in chicks under white light conditions. At 13 days of exposure, eyes with lens wear had shorter (**a**) VCD and (**b**) AL than the contralateral control eyes. **c, d** Refraction in lens-wearing eyes were more hyperopic than the control eyes. Data represents mean ± SEM (n = 8 per condition). *Statistically significant at *P* less than 0.05 (one-way repeated-measures ANOVA with Tukey HSD) when compared with control eyes. VCD, vitreous chamber depth; AL, axial length; SER, spherical equivalent refraction; SEM, standard error of mean

The effect of opposing dual-power lenses on ocular growth was assessed in chicks raised under white light conditions by comparing eyes with and without lenses. After 13 days of lens wear, eyes wearing dual-power lenses grew significantly less when compared with contralateral control eyes without lenses (at D13, change in VCD, treated *vs.* control: 0.54 ± 0.08 mm *vs.* 0.81 ± 0.04 mm, *P* < 0.01, Fig. 4a; change in AL, treated *vs.* control: 1.25 ± 0.04 mm *vs.* 1.45 ± 0.05 mm, *P* < 0.01, Fig. 4b). Refraction in the eyes treated with lenses became more hyperopic when compared to the control eyes (at D13, change in SER, treated *vs.* control: 1.09 ± 0.51 D *vs.* − 1.80 ± 0.27 D; *P* < 0.001, Fig. 4c; mean SER, treated *vs.* control: 4.81 ± 0.43 D *vs.* 1.77 ± 0.21 D, *P* < 0.001, Fig. 4D). It was consistent with the observation that dual-power lenses induced an intermediate response between the two constituent powers, with a greater weight towards myopic defocus, to emmetropic animals [22, 23].

### Effect of narrowband light on eye growth

**Fig. 5:**
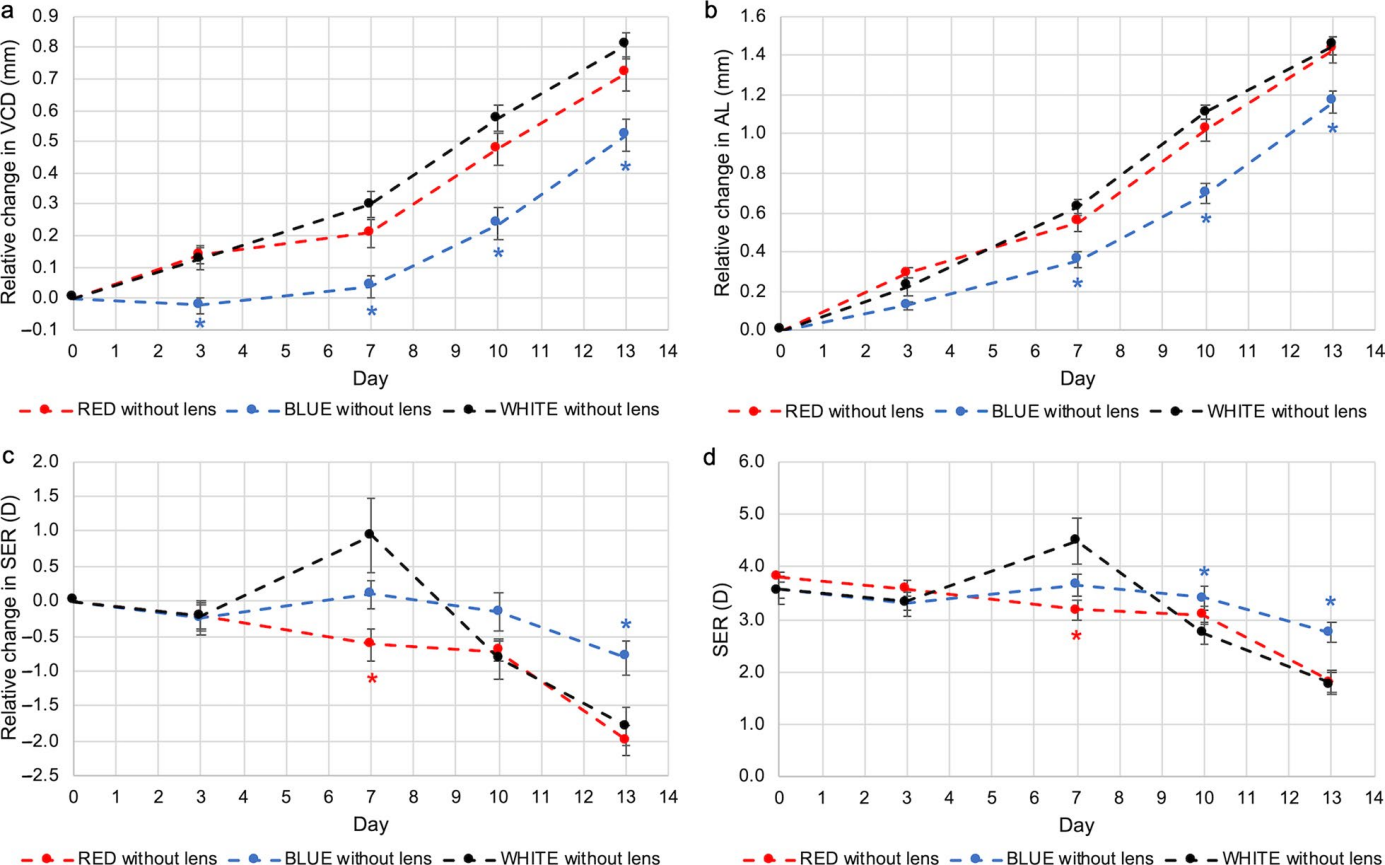
Effect of narrowband light on eye growth in control eyes under different light conditions. Control eyes exposed to blue light showed significantly shorter (**a**) VCD and (**b**) AL, and more hyperopic refraction **c, d**. No significant differences between red and white light were observed. Data represents mean ± SEM (n = 8 per condition). * Statistically significant at *P* less than 0.05 (one-way repeated-measures ANOVA with Tukey HSD) when compared to chicks exposed to white light. VCD, vitreous chamber depth; AL, axial length; SER, spherical equivalent refraction; SEM, standard error of mean

Narrowband blue light modulated ocular growth in the absence of any lens wear. Starting from D3, VCD in control eyes was significantly shorter in chicks raised in blue light condition relative to white light condition, and it remained significantly shorter throughout the duration of the experiment up to D13 (at D13, change in VCD, blue *vs.* white: 0.52 ± 0.05 mm *vs.* 0.81 ± 0.04 mm, *P* < 0.001, Fig. 5a). A similar pattern was observed for AL, except that a statistically significant difference versus white light was only observed starting from D7, while the difference on D3 did not reach statistical significance (Fig. 5b). At D13, AL was significantly shorter in chicks raised in blue light condition (change in AL, blue *vs.* white: 1.17 ± 0.06 mm *vs.* 1.45 ± 0.05 mm, *P* < 0.001, Fig. 5b). No significant difference was seen between chicks raised in white and red light conditions in VCD or AL at any time point.

After 13 days of exposure, refractive errors of control eyes exposed to blue light were significantly more hyperopic compared to those exposed to white light (at D13, change in SER, blue *vs.* white: − 0.81 ± 0.25 D *vs.* − 1.80 ± 0.27 D, *P* < 0.05, Fig. 5c; mean SER, blue *vs.* white; 2.75 ± 0.21 D *vs.* 1.77 ± 0.21 D, *P* < 0.05, Fig. 5d). No significant difference in refraction was seen between eyes exposed to red and white light except on D7 (at D7, change in SER, red *vs.* white: − 0.63 ± 0.24 D *vs.* 0.94 ± 0.52 D, *P* < 0.05, Fig. 5c; mean SER, red *vs.* white: 3.19 ± 0.19 D *vs.* 4.50 ± 0.45 D, *P* < 0.05, Fig. 5d).

### Combination effect of dual-power lenses and narrowband lighting on eye growth

**Fig. 6:**
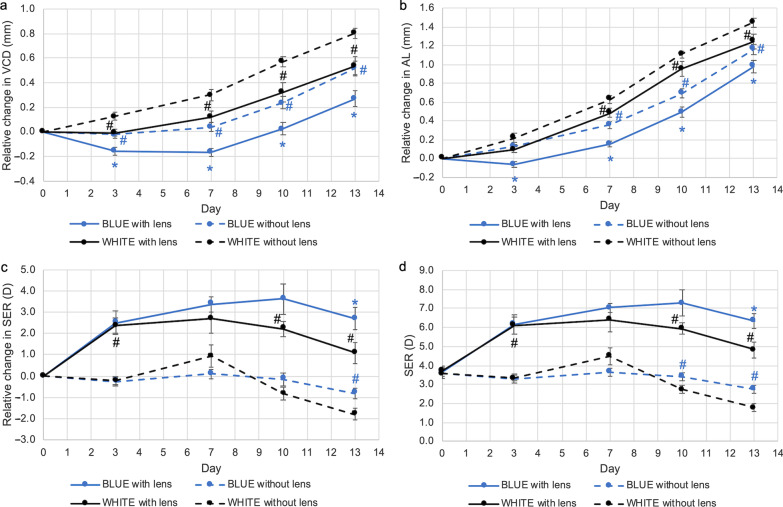
Combination effect of dual-power lenses and narrowband blue light on eye growth. Blue light significantly further reduced (**a**) VCD and (**b**) AL beyond the effect mediated by dual-power lenses under white light conditions. **c, d** At Day 13, refraction of lens-wearing eyes under blue light remained significantly more hyperopic than that under white light. Data represents mean ± SEM (n = 8 per condition). *^,#^Statistically significant at *P* less than 0.05 (one-way repeated-measures ANOVA with Tukey HSD) when compared with control eyes (^#^) or lens-wearing eyes (*) exposed to white light. VCD, vitreous chamber depth; AL, axial length; SER, spherical equivalent refraction; SEM, standard error of mean

**Fig. 7:**
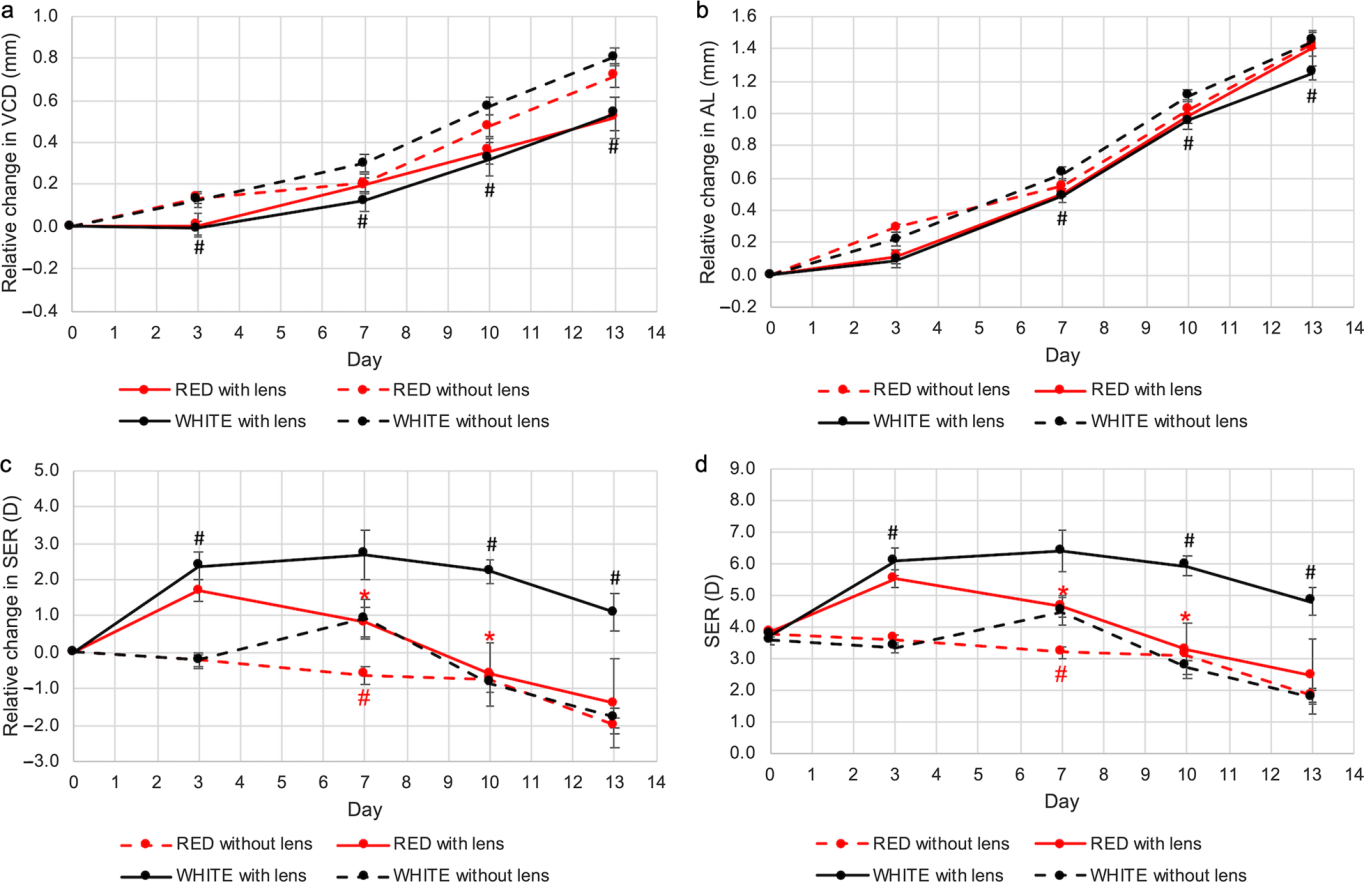
Combination effect of dual-power lenses and narrowband red light on eye growth. Red light had no effects on (**a**) VCD and (**b**) AL beyond the effect mediated by dual-power lenses under white light conditions. **c, d** At Day 7 and Day 10, refraction of lens-wearing eyes under red light was significantly less hyperopic than that under white light. Data represents mean ± SEM (n = 8 per condition). *^,#^Statistically significant at *P* less than 0.05 (one-way repeated-measures ANOVA with Tukey HSD) when compared with control eyes (^#^) or lens-wearing eyes (*) exposed to white light. VCD, vitreous chamber depth; AL, axial length; SER, spherical equivalent refraction; SEM, standard error of mean

VCD and AL in eyes wearing dual-power lenses changed differently between the three lighting conditions. For both parameters, eye growth was inhibited in lens-wearing eyes exposed to blue light relative to white light at all time points (*P* < 0.05, Fig. 6a and b). Remarkably, VCD on D3 and D7 and AL on D3 shortened relative to baseline in lens-wearing eyes exposed to blue light. At D13, the eyes exposed to blue light plus dual-power lens had the shortest VCD and AL (change in VCD, blue *vs.* white: 0.27 ± 0.07 mm *vs.* 0.54 ± 0.08 mm, *P* < 0.05, Fig. 6a; change in AL, blue *vs.* white: 0.99 ± 0.05 mm *vs.* 1.25 ± 0.04 mm, *P* < 0.05, Fig. 6b). Red light exposure had no significant effect on VCD and AL in comparison to animals reared under white light (Fig. 7a and b).

Refractive errors remained hyperopic in eyes exposed to dual-power optical lenses under all conditions throughout the entire study period (Figs. 6c and 7c). However, relative to white light conditions, refraction in lens wearing eyes exposed to blue light remained the most hyperopic (Fig. 6c and d), whereas eyes exposed to red light were the least hyperopic (Fig. 7c and d). The difference between blue and white light was statistically significant on D13 (change in SER, blue *vs.* white: 2.70 ± 0.53 D *vs.* 1.09 ± 0.51 D, *P* < 0.05, Fig. 6c; mean SER, blue *vs.* white: 6.36 ± 0.39 D *vs.* 4.81 ± 0.43 D, *P* < 0.001, Fig. 6d), while the difference between red and white light was significant on D7 and D10 but did miss significance on D13. Notably, no significant difference in SER was observed between control eyes in white light and lens-wearing eyes in red light conditions after D3, demonstrating that red light exposure almost entirely negated the effect of the dual-power lens (Fig. 7c and d).

### Combination effect of dual-power lenses and narrowband lighting on choroidal thickness

**Fig. 8:**
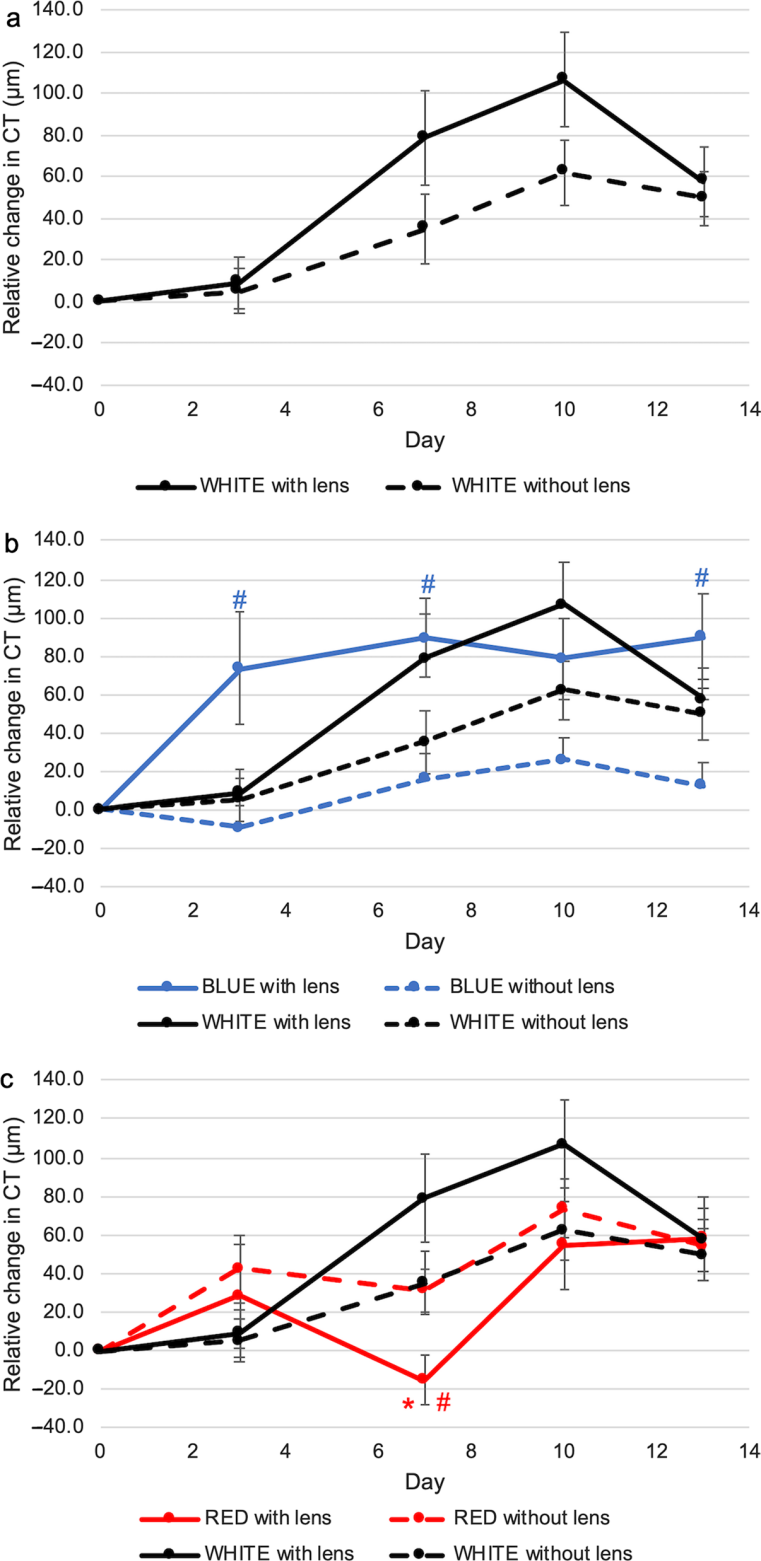
Combination effect of dual-power lenses and narrowband light on choroidal thickness (CT). **a** Lens-wearing alone had no significant effect on CT. **b** Blue light exposure induced a significant choroidal thickening in lens-wearing eyes but not in control eyes. **c** Red light exposure induced a significant choroidal thickening in both lens-wearing and control eyes starting from Day 10. Data represents mean ± SEM (n = 8 per condition). *^,#^Statistically significant at *P* less than 0.05 (one-way repeated-measures ANOVA with Tukey HSD) when compared to contralateral control eyes exposed to corresponding narrowband light (^#^) or lens-wearing eyes (*) exposed to white light. SEM, standard error of mean

Consistent with CT being an early/predictive indicator of eye growth [57], choroid thickened significantly starting from D10 in control eyes exposed to white light when compared with D0 (at D13, change in CT = 49.77 ± 13.35 µm, *P* < 0.01, Fig. 8a). 13-day wearing of dual-power lens had no significant effect on CT under white light conditions (Fig. 8a). Under blue light exposure, lens-wearing eyes had a significant choroidal thickening throughout the entire study period (at D13, change in CT = 89.60 ± 22.26 µm, *P* < 0.01, Fig. 8b) while the control eye had no significant change in CT when compared with D0. The IOD between the change in CT relative to baseline was greater in eyes under blue light than under white light and the differences were significant on D3 and D13 (at D13, IOD for change in CT, blue *vs.* white: 77.33 ± 22.83 µm *vs.* 8.04 ± 21.05 µm, *P* < 0.05, Additional file 1). No significant difference in CT was seen in lens-wearing chicks raised in blue and white conditions (Fig. 8b).

Red light exposure induced a significant choroidal thickening in control eyes throughout the entire study period when compared with D0 (at D13, change in CT = 54.33 ± 13.59 µm, *P* < 0.01, Fig. 6c). Lens-wearing had no significant effect on choroidal thickening when exposed to red light, except on D7 (at D7, change in CT, treated *vs.* control: − 15.50 ± 12.79 µm *vs.* 31.21 ± 11.08 µm, *P* < 0.01, Fig. 8c). Notably, both lens-wearing and control eyes had less choroidal thickening at D7 in red light condition. At D7, the IOD between the change in CT was significantly smaller in eyes exposed to red light when compared with that in white condition (at D7, IOD for change in CT, red *vs.* white: − 46.71 ± 12.30 µm *vs.* 43.92 ± 31.97 µm, *P* < 0.05, Additional file 1).

### Associations between choroidal thickness and axial length or refraction

**Fig. 9:**
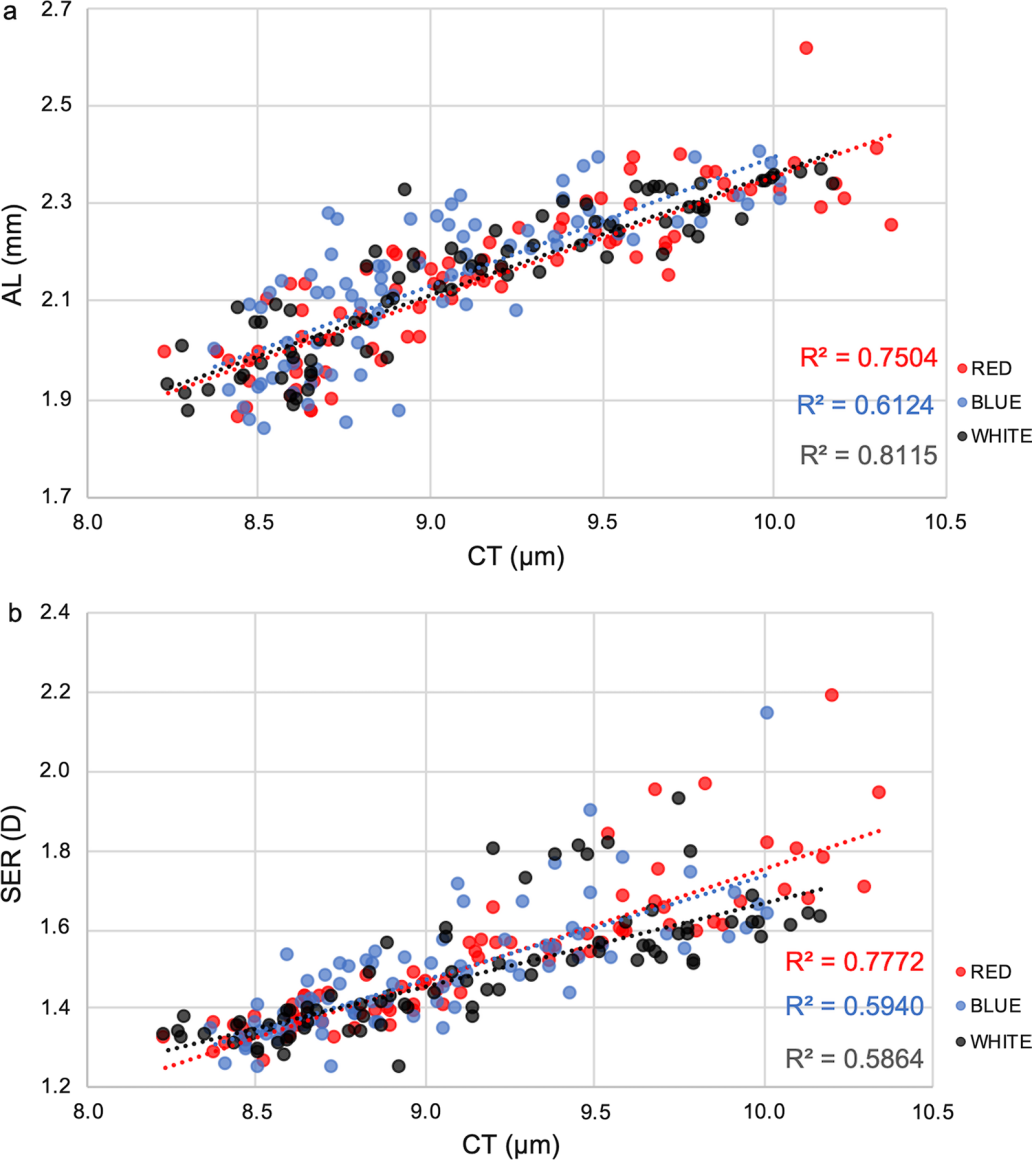
Associations between choroidal thickness (CT) and axial length (AL) or refraction. **a** Associations between CT and AL in eyes under red, blue or white conditions throughout the entire study period; **b** Associations between CT and refraction (as SER) in eyes under red, blue or white conditions throughout the entire study period. SER, spherical equivalent refraction

CT had significant, strong and positive correlations with AL in both eyes of chicks reared in all light conditions throughout the entire study period (all *P* < 0.05, Fig. 9a and Table 2). CT was also strongly and positively associated with refraction error in both eyes under all light conditions (all *P* < 0.05, Fig. 9b and Table 2).

**Table 2:** Associations between choroidal thickness and axial length or refraction in chicks throughout the study period

	Pearson coefficient	R square	*P* value
CT *vs.* AL			
Red	0.882	0.750	0.004
Blue	0.771	0.612	0.025
White	0.766	0.812	0.027
All	0.810	0.655	0.015
CT *vs.* SER			
Red	0.866	0.777	0.005
Blue	0.783	0.594	0.022
White	0.901	0.586	0.002
All	0.847	0.717	0.008

*CT* = choroidal thickness; *AL* = axial length; *SER* = spherical equivalent refraction

## Discussion

Several studies have investigated how developmental eye growth and refraction are affected by light parameters, including light intensity, photoperiod, and wavelength spectrum distribution [43, 44, 58–62]. In this study, we examined the effect of light conditions (white, red, and blue) combined with opposing dual-power optic defocus on eye growth and refraction. Individually, both narrowband blue light and dual-power lens induced a hyperopic shift and reduced axial elongation in chicks. When combined, the effect was greatly enhanced. The eyes exposed to narrowband blue light plus dual-power lens had the least axial elongation (change in AL = 0.99 ± 0.05 mm) and were the most hyperopic (mean SER = 6.36 ± 0.39 D) after 13 days of light exposure. To the best of our knowledge, this study is the first to demonstrate additive effects of narrowband light and optical defocus on eye growth in chicks.

The effect of the dual-power lens on SER under white light conditions here is consistent with effects previously published and is less effective than a + 10 D single vision (SV) lenses [22]. However, unlike single vision positively powered lenses, a dual-powered lens is a reasonable approximation of defocus incorporated multiple segments (DIMS) lenses that are used to reduce myopia in schoolchildren [27]. DIMS lenses are corrective lenses that contain alternating positively and negatively powered segments in the periphery of the visual field. This creates a defocus stimulus without affecting central vision. DIMS lenses impose myopic defocus in a controlled clinical study [24, 26, 27] but did not completely halt myopia progression. Full-time wearing of (15.5 ± 2.6 h/day) DIMS lenses with + 3.5 D defocus slowed myopia progression by 52% and axial elongation by 62% over a two-year period when compared with SV lens wearing [27]. The effect was sustained in the third year and that myopia progression was slowed by 86% and axial elongation was slowed by 61% [24]. Combining DIMS lenses with other treatment methods to more effectively reduce myopia progression is of great interest. Ideally, this combination treatment should not negatively impact the favorable safety profile of DIMS lenses. One promising strategy is the manipulation of light chromaticity. However, before experimental treatments are tested in clinical studies involving schoolchildren, it is necessary to have preclinical evidence of additive efficacy. This study is a first step in this direction, as it shows additive efficacy when the dual-power lens is combined with blue light exposure.

Interestingly, while red light exposure alone did not affect eye growth or refraction, it partially countered the effects of the dual-power lens on refraction and CT. These results suggest that the effects of hyperopic defocus are influenced by light chromaticity, with shorter wavelength (blue) light enhancing the effects and longer wavelength (red) light partially counteracting them. The IOD of visual parameters measures the effect of the dual-power lens under consistent light conditions. The IOD between change in refraction relative to baseline under red light conditions was smaller than under white light conditions, further demonstrating an interaction between wavelength and optical defocus. It is worth noting that the IOD between change in refraction under blue light conditions was numerically higher than under white light conditions, suggesting the possibility of a synergistic effect between blue light and optical defocus. However, more studies with larger sample sizes are needed to confirm this. It is important to note that light intensity was controlled and identical between all three light conditions in this study, so it did not contribute to the group differences observed.

It has been demonstrated that the choroid assists emmetropization by changing its thickness to move the retina forward or backward in response to imposed myopic or hyperopic defocus [55]. Control eyes induced choroidal thickening in response to rapid eye growth and natural hyperopic shift under white condition. Under blue light exposure, the choroid significantly thickened in lens-wearing eyes when compared with control eyes, in response to the significant hyperopic shift induced by the combinatorial effect of dual-power lenses and narrowband blue light. Under red light exposure, there was a sudden choroidal thinning in lens-wearing eyes at D7 which is seen in response to the myopic shift induced. CT had significant associations with both AL and SER, indicating that the choroid plays an important role in emmetropization and CT is an early/predictive indicator of eye growth [57].

The role of spectral composition of ambient light in emmetropization to regulate natural ocular growth and refractive development has been demonstrated in chicks. A myopic shift in chick eyes with increased axial elongation upon red light exposure has been observed [58, 59, 63, 64]. In contrast, short-wavelength, blue light exposure might induce hyperopia and provide protective effect against myopia progression in chicks [44, 60, 62, 65]. Similar hyperopic shift and protective effect against myopia were also observed in chicks [53] exposed to short-wavelength, violet light. Despite some inconsistency in the findings from red light rearing chicks, our results were in tandem with other studies where blue light rearing can induce hyperopia in chicks.

There are currently no clinical studies on the combination effect of colored light and optical defocus. However, clinical trials have been conducted to study the effect of stand-alone light components using light transmitting or filtering lenses on myopia control [53, 54, 66]. Daily wear of blue-violet light filtering spectacle lens showed no significant effect on myopia progression and eye growth over a one-year period [66], indicating that the myopia control effect of blue light is less pronounced in humans than in animals. Conversely, daily wear of violet light transmitting spectacle lens showed significant reduction in axial elongation in children over a one-year period [53]. In addition, the implantation of violet light transmitting phakic intraocular lens significantly slowed axial elongation in adults with high myopia (less than − 10 D) over a five-year period [54]. While a consensus on clinical myopia control effect with blue light is yet to be reached, the above clinical observations are consistent with the animal studies [53, 67, 68] where violet light may also play an important role in slowing myopia development.

Overall, the results of this study support exploring the effects of wavelength modulation for the treatment of myopia, including in combination with optical defocus. However, in a clinical setting, blue light illumination as used in this study is impractical as increased exposure to short-wavelength light has been shown to disrupt circadian rhythm and raise health concerns [69–75]. Increased use of artificial light enriched with short-wavelength (around 480 nm) stimulates intrinsically photosensitive retinal ganglion cells [70, 73] and suppresses night-time melatonin levels [74, 75], which, in turn, affects sleep quality [74, 75] and may confer a high risk for cancers [69, 72]. Therefore, the use of lenses with wavelength filters is a more realistic method for modulating light chromaticity in the clinic. Further preclinical studies using defocus lenses with wavelength filters are needed before conducting clinical studies. One challenge in using wavelength filters is ensuring consistent light intensity between treatment groups, as the filters may also reduce light intensity, which can complicate the interpretation of study results. The results of this study provide a benchmark that is not influenced by group differences in light intensity, allowing for comparison with future studies using different methods of modulating chromaticity. There are some potential limitations in this study. Chicks have different properties and spectral sensitivity of photoreceptors from that of humans. For instance, human color vision is trichromatic [76] while chicks are tetrachromatic [77], with an extra double cone [78]. Hence, these limit a direct extrapolation of results from our study to human. In addition, chicks have different spectral sensitivities under different light conditions. They are more sensitive to red than blue light [79]. With our experimental setup, we were unable to adjust the irradiance of our LED lights to allow chicks to receive equalized illuminance under different light conditions. Due to the spectral sensitivity, chicks might receive a comparatively lower illuminance under blue light than red light. As dim light promotes myopia progression [80], the hyperopic shift observed in eyes under blue light may be underestimated. Moreover, we did not include chicks with both eyes untreated as naïve control. It has been proposed that both light conditions and optical defocus can induce compensation responses in contralateral untreated eyes and affect eye growth [10, 80]. A naïve control will help monitor the eye growth under normal conditions and clarify the yoking effect and consensual responses caused by light conditions and optical defocus.

Despite the hyperopic shift in chicks under narrowband blue light, we did not find the expected opposite response in red light seen by others [44, 58, 63]. Our results showed that untreated eyes under red light exposure had comparable refractive development and axial elongation as those under white light exposure. However, we did not investigate the role of cornea in emmetropization in this study. It has been proposed that corneal curvature and thickness are sensitive to photoperiod and light intensity [81–83]. Long-term exposure to light, particularly long wavelengths, caused corneal flattening and a hyperopic shift in chicks, which may compensate for the refractive effect of vitreous chamber elongation [84, 85]. This may explain the hyperopic refraction in eyes under all light conditions, despite significant eye growth. As the eyes remained hyperopic, the magnitude of refractive error decreased over the study period, suggesting the need for further research on the contribution of the cornea to refractive development and eye growth under different light conditions and optical defocus.

## Conclusions

Our results indicated that the spectral composition of light plays a significant role in modulating eye growth. Both narrowband blue light and dual-power lens interventions induced a hyperopic shift and reduced axial elongation in chicks, providing protection against myopia development. The effect of these interventions was additive. These findings support the use of optical defocus interventions combined with wavelength filters in clinical studies testing their effectiveness in treating myopia in children [53, 66].

## Supplementary Information

**Additional file 1: Table S1. **The refraction and ocular parameters in eyes of chicks under all conditions throughout the study. A summary table showing an overview of all the statistical results obtained throughout the study.

## Data Availability

Not applicable.

## Abbreviations

ACD: Anterior chamber depth
AL: Axial length
B: Blue
BC: Blue without lens
BT: Blue with lens
ChS: Choroidal-scleral
CT: Choroidal thickness
D0: Pre-exposure
D3: Day 3
D7: Day 7
D10: Day 10
D13: Day 13
DIMS: Defocus incorporated multiple segments
IOD: Interocular difference
LED: Light emitting diode
LT: Lens thickness
PMMA: Polymethylmethacrylate
R: Red
RC: Red without lens
RCh: Retina-choroidal
RT: Red with lens
S: Sclera
SEM: Standard error of mean
SER: Spherical equivalent refraction
SV: Single vision
VCD: Vitreous chamber depth
VR: Vitreal-retinal
W: White
WC: White without lens
WT: White with lens
